# Myricetin Restricts the Syncytial Development Triggered by Nipah Virus Envelope Glycoproteins In Vitro

**DOI:** 10.3390/v17060827

**Published:** 2025-06-07

**Authors:** Ananda Murali Rayapati, Chanda Chandrasekhar, Sudarsana Poojari, Bhadra Murthy Vemulapati

**Affiliations:** 1 Koneru Lakshmaiah Education Foundation, Vaddeswaram, Guntur 522502, India; muralirayapati@gmail.com (A.M.R.); chandrasekharchanda02@gmail.com (C.C.); 2 Cool Climate Oenology and Viticulture Institute, Brock University, St. Catharines, ON L2S 3A1, Canada; spoojari@brocku.ca

**Keywords:** antivirals, myricetin, Nipah virus, F and G envelope glycoproteins, syncytia, in vitro assay

## Abstract

*Background and Objectives:* Myricetin, a flavonoid compound, was demonstrated to effectively arrest the cell-to-cell fusion and syncytial development triggered by Nipah virus (NiV) fusion (F) and attachment (G) envelope glycoproteins in vitro involving two permissive mammalian cell lines. *Methods:* Time-of-addition assays were carried out using codon-optimized NiV wild type (WT) F and G plasmids followed by a challenge with the addition of myricetin 1 h and 6 h post-transfection in HEK 293T and Vero cells. *Results:* Upon evaluating different myricetin concentrations, it was determined that a 100 μM concentration of myricetin effectively inhibited 64–80% of syncytia in HEK and Vero cells. *Interpretation & Conclusions:* In this study, we concluded that myricetin mitigated the syncytial development in HEK and Vero cell lines. Given the flavonoid attributes of myricetin which is widely present in fruits, vegetables, tea, and wine, it may be regarded as a phytonutrient and a safer antiviral alternative against Nipah virus infections. Due to the BSL-4 nature of the virus, further research involving live virus culture is necessary to confirm myricetin as a potential antiviral compound for the mitigation of pathological effects of NiV infections.

## 1. Introduction

Nipah virus (NiV) is an emergent and highly pathogenic zoonotic paramyxovirus in the genus Henipavirus. NiV first emerged in Malaysia and Singapore in 1999. NiV was associated with an outbreak of severe febrile encephalitis associated with human deaths reported in Peninsular Malaysia beginning in late September 1998 [1]. Later, NiV outbreaks led to several outbreaks since 2001 in Bangladesh, India, Singapore, Malaysia, and other countries [2]. NiV targets vascular endothelial and neuronal cells causing severe encephalitic or respiratory syndrome with case fatality rates ranging from 40 to 100% in humans [2,3,4]. Due to its high mortality, ease of transmission between species, and lack of effective therapy or prophylaxis, NiV is categorized as a biosafety level 4 (BSL-4) pathogen. NiV requires the coordinated action of envelope glycoproteins G and F to recognize, bind, trigger, and gain entry into susceptible cells. NiV binds to ephrin-B2 or -B3 receptors that are expressed on microvascular endothelial cells to gain entry [5,6,7,8]. NiV glycoprotein G is responsible for the receptor binding resulting in conformational changes in G and triggering F glycoprotein. After triggering, the pre-fusion F will be activated to a post-fusion F that harpoons itself into the adjacent cells causing the development of syncytium. Syncytia represent a cellular structure formed due to multiple cell fusions of individual mononuclear cells [5,9,10]. Endothelial syncytium formation is a peculiar hallmark of NiV infection leading to cell destruction, inflammation, and hemorrhage. The syncytium leads to further spread, inflammation, and damage to endothelial cells and neurons [11]. As a result, NiV is listed in the World Health Organization (WHO) R&D Blueprint list of priority pathogens [12]. Since 2001, India witnessed six Nipah virus outbreaks, and the first outbreak was reported in the state of West Bengal with a case fatality (CFR) of 68%. Subsequently, outbreaks have been reported from West Bengal and Kerala with high CFR [13]. To date, only limited data are available on NiV treatment strategies due to the pathogenic nature of the virus and experiments requiring BSL-4 facilities. No approved vaccines or treatments that specifically target NiV are available, and quarantine has been the predominant measure to limit the spread of the virus [14].

Substantial work has been carried out on the role of flavonoids as potential antivirals against several animal and human viruses, including COVID-19. Flavonoids were shown to exhibit their antiviral activity by (i) hindering the viral entry; (ii) interfering with the host receptor binding; (iii) inhibiting the function of the host-receptor itself; (iv) hampering the viral replication process and/or viral assembly; (v) blocking the release and cell-to-cell movement; and (vi) enhancing immune responses [15,16,17]. Myricetin (MYR) is a flavonoid compound found in vegetables, fruits, and tea and possesses antioxidant properties [18,19,20], as well as antimicrobial, anti-thrombotic, neuroprotective, and anti-inflammatory properties [21,22,23]. MYR has been shown to exhibit antiviral properties on several pathogenic viruses which include HIV, HSV, SARS, and SARS-CoV-2 [24,25,26,27,28,29,30,31]. In this study, the therapeutic potential of MYR as an antiviral agent and its role in mitigating the syncytial development triggered by NiV-F and -G envelope glycoproteins in vitro was investigated.

## 2. Materials and Methods

### 2.1. Cell Lines and Reagents

**Figure 1:**
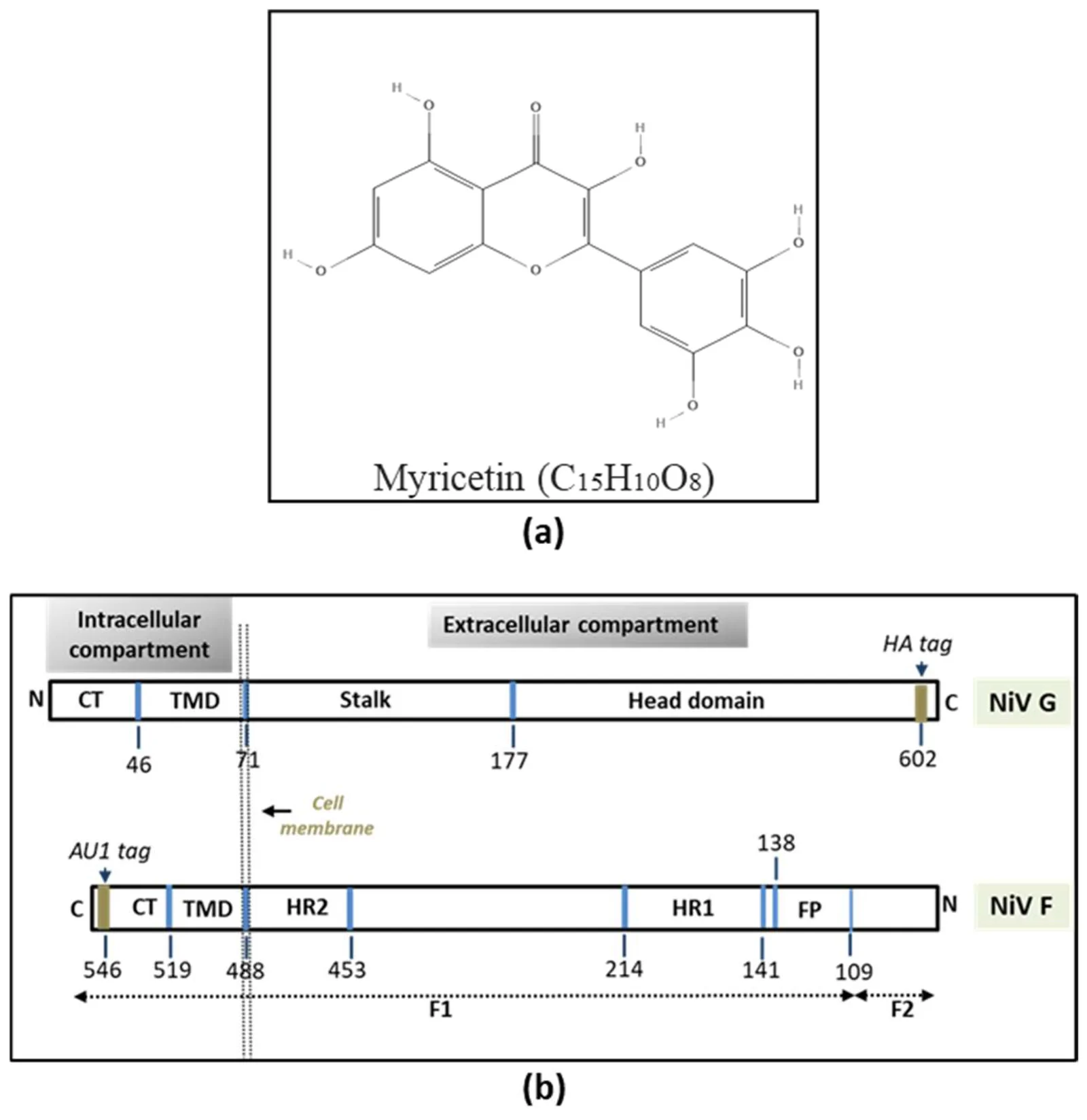
(**a**) Structure of myricetin (MYR; 3,5,7-trihydroxy-2-(3,4,5-trihydroxyphenyl) chromen-4-one) also known as cannabiscetin, a bioflavonoid evaluated in this study. URL: https://pubchem.ncbi.nlm.nih.gov/compound/5281672#section=Structures (accessed on 12 January 2020) (**b**) Codon-optimized wild-type (WT) Nipah virus attachment glycoprotein G (NiV-G) and fusion glycoprotein F (NiV-F). The components of F and G plasmids and their amino acid positions are represented. The F and G detection tags (HA/AU1) are highlighted in the plasmids. The cellular positions of F and G with respect to the intracellular and extracellular compartments are represented by a dotted line across the plasmid organization.

The cell lines used in this study were purchased from the National Center for Cell Sciences (NCCS, Pune, India). Two NiV permissive mammalian cell lines (expressing ephrin-B2 receptor on the cell surface) and a non-permissive cell line (very few ephrin-B2) were used in this study. The NiV permissive cell lines were (i) human embryonic kidney cells (HEK293T) and (ii) African green monkey kidney epithelial cells (Vero). The non-permissive cell line used in this study was the Chinese hamster ovary (CHO) cell line. HEK293T cells were cultured in Dulbecco’s modified Eagle medium (DMEM) (Gibco, USA), while CHOs and Vero cells were cultured in minimal essential medium (MEM) (Gibco, USA), both supplemented with 10% fetal bovine serum (FBS; Thermofisher Scientific, San Diego, CA, USA), 50 IU of penicillin ml-1 (Sigma-Aldrich, St. Louis, MO, USA), streptomycin ml-1 (Sigma-Aldrich, St. Louis, MO, USA), and 2 mM glutamine (Sigma-Aldrich, St. Louis, MO, USA). Myricetin (3,3′,4′,5,5′,7-Hexahydroxyflavone) MW-318.23 g/mol (Figure 1a) purchased from the Tokyo Chemical Industry Co., Ltd. (TCI, India) was used in all the in vitro assays carried out in this study. MYR stock solution was prepared according to the manufacturer’s instructions. Seven testing concentrations of MYR (10, 25, 50, 100, 250, 500, and 1000 μM) were evaluated in the study to assess the antiviral potential of MYR.

### 2.2. Plasmid Constructs

Codon-optimized wild-type (WT) Nipah virus attachment G (NiV-G) and fusion glycoprotein F (NiV-F) were synthesized in pcDNA3.1 vector construct (GenScript, USA). The NiV-G was tagged at the C-terminus with the hemagglutinin (HA) tag and the NiV-F was tagged at the C-terminus with the AU1 tag (Figure 1b). The plasmid construct design and organization were reported earlier [5]. The NiV-G encodes a 602 amino acid attachment glycoprotein (~1.8 kb nucleotides), and the NiV-F encodes a 546 amino acid (~1.6 kb nucleotides) fusion glycoprotein, respectively. The inclusion of tags in the WT F and G plasmids facilitates the detection of expressed viral proteins using SDS-PAGE and Western blotting with tag-specific primary and secondary antibodies.

### 2.3. Confirmation of NiV F and G Plasmids

The lyophilized pcDNA3.1 vector constructs encoding the wild type (WT) F and G were suspended in TE buffer and used for the transformation of E. coli DH5-α competent cells following standard molecular biology protocols [32]. Following blue-white screening, F and G plasmid DNA was isolated from the bacterial cultures using a Plasmid mini kit (Qiagen, Germany) according to the manufacturer’s instructions. The confirmation of the F and G sequence in the pcDNA3.1 vector was carried out by polymerase chain reaction (PCR) using a thermal cycler (Eppendorf, Germany) with forward (CMV-Fwd) and reverse (BGH-Rev) primers flanking the multiple cloning site (MCS). The resulting PCR products were confirmed by electrophoresis on 1% agarose gels.

### 2.4. MTT (Cell Viability) Assay

Cell viability (cytotoxicity) of MYR on the three cell lines used in this study was carried out using the MTT assay (EZcount™ MTT Cell Assay Kit, Himedia, India) in 96-well immunoplates (Nunc, San Diego, CA, USA) according to the manufacturer’s instructions. Different concentrations of MYR (10, 25, 50, 100, 250, 500 and 1000 μM) were tested on the three cell lines to determine cytotoxicity. Briefly, cells at 70% confluency were treated with MYR diluted in DMEM (100 μL per well). Plates were incubated at 37 °C in biological CO2 incubator (5%) for 24 h. The multi-well plates were treated with 15 μL of dye for the MTT assay and incubated for 4 h. Later, the cell viability (cytotoxicity) was determined by spectrophotometric analysis at 570 nm using a microplate reader (BioTek μQuant Microplate Spectrophotometer, Watertown, MA, USA). The viability of MYR-treated cells was calculated as a percentage relative to values obtained with DMSO-treated cells.

### 2.5. Transfection with F and G Plasmids

NiV-F and -G plasmids (2:1 ratio) were transfected into HEK293T or Vero cells at ~70% confluence at 2 µg/well in 6-well plates. The concentration of the F and G plasmids used for transfection in 12- or 24-well plates was adjusted accordingly. Different combinations of NiV F and G plasmids were included in the study to determine the progress and inhibition of syncytia in permissive and non-permissive cell lines. The different transfection combinations were (i) NiV-F only (F+pC3.1); (ii) NiV-G only (G+pC3.1); and (iii) NiV F+G (co-transfection). Positive controls were NiV F+G (co-transfected) without MYR treatment, and negative controls consisted of only pCDNA3.1 transfected cells. CHO cells were included as an additional negative control in all the experiments (Supplementary Table S1). In cells that were transfected only with F or G alone, the final plasmid DNA concentration in each well was adjusted with pcDNA3.1. Briefly, on Day 0, the 6-well plates were seeded separately with HEK293T, VERO, or CHO cells (~2 × 10^6^), respectively. The growth medium used was either DMEM or MEM supplemented with 10% FBS. After the cells reached 70% confluence on Day 1, cells were transfected with NiV-F and(or) -G plasmids using Lipofectamine-3000 transfection reagent (ThermoFisher Scientific, USA) according to the manufacturer’s instructions. Before the cells were fixed or treated with different concentrations of MYR, the growth medium was aspirated with an automatic aspirator, and the cells were washed once with phosphate-buffered saline supplemented with 1% FBS.

### 2.6. Time-of-Addition (Time-Course) Assay of Antivirals

Time-of-addition experiments were designed to investigate the antiviral potential of MYR on syncytial (cell-to-cell fusion) progression in HEK293T, Vero, and CHO cells at two different stages after the completion of transfection. MYR was diluted to the required concentrations in MEM or DMEM supplemented with 10% FBS and added to the cells with different combinations of F and G plasmids (Supplementary Table S1). MYR was added to the cells at 1 h and 6 h post-transfection. The cells were washed once with PBS containing 1% FBS before the addition of MYR diluted in the growth medium. All the experiments were carried out in 6-well plates (Nunc, San Diego, CA, USA) unless specified. Each of the MYR concentrations was tested in triplicate to assess effective inhibitory concentration.

### 2.7. Quantification of Syncytia

Syncytial development, progression, and quantification in the multi-well plates treated with different concentrations of MYR were observed using a phase-contrast microscope (Olympus-CKX53, Japan) 18 h post-transfection. Ten fields per well of 6-well plates were observed under 100X magnification for the quantification of syncytia. A total of 30 fields were observed for each MYR treatment. Wells transfected with NiV F+G only (without MYR) were considered as positive control, while cells transfected with (i) pCDNA3.1 only; (ii) F only; and (iii) G only were considered as negative controls. CHO cells (non-permissive) were included as an additional negative control. Then, 18–24 h after the addition of MYR, the cells were fixed with 0.5% paraformaldehyde (PFA) to halt further syncytial development in the wells and to facilitate quantification of syncytia. The percentage of syncytial inhibition was measured as the reduction in the number of syncytia in each well in comparison to the positive and negative controls.

### 2.8. Protein Expression, SDS-Polyacrylamide Gel Electrophoresis (PAGE), and Western Blotting

To determine if MYR could block or interfere with the expression of NiV G and F in HEK293T cells, SDS-polyacrylamide gel electrophoresis (PAGE) [33] was carried out followed by Western blotting. The non-transfected, transfected (without treatment with MYR), and antiviral-treated cells were harvested 18 h after treatment with MYR in 6-well plates. Following transfection with NiV F and G plasmids, the cells were harvested from each well using cell lysis buffer (Genei, India) according to the manufacturer’s instructions. Equal volumes of the protein lysate were loaded on 12% PAGE gels containing SDS, followed by membrane transfer onto a nitrocellulose membrane (NCM) for probing with antibodies against F and G glycoproteins. Primary and secondary antibodies were used for the detection of expressed F and G glycoproteins in transfected cells by Western blotting. NiV-G glycoprotein was detected with an anti-HA tag antibody (the primary antibody was rabbit anti-HA antibody (1:500–1000 dilution) while the secondary antibody was goat anti-rabbit antibody (1:1000–2000 dilution)) (Thermofisher Scientific, USA). The F protein was detected using an anti-AU1 antibody (primary antibody was goat anti-AU1 polyclonal antibody (1:1000–2000 dilution) and the secondary antibody used was rabbit anti-goat antibody (1:1000–5000 dilution)) (Thermofisher Scientific, USA). The band intensities from the same volume of each lysate were determined.

## 3. Results

### 3.1. Confirmation of NiV-F and G Plasmids

Each of the transformed recombinant F and G DH5α colonies were picked, and plasmid DNA isolation was carried out. PCR was performed using F and G plasmid DNA, and PCR products were analyzed on 1% agarose gels. PCR products yielded the expected size of F and G sequences. The NiV-F insert yielded ~1.6 kilo basepairs, and the NiV-G insert generated ~1.8 kilo basepairs, respectively (Supplementary Figure S1A).

### 3.2. MTT Assay

Of the seven different MYR concentrations tested, a mild cytotoxicity of ~10–15% was exhibited in 250 μM; 70–80% in 500 μM, and 100% cytotoxicity was observed in 1000 μM in all the three cell lines tested. The remaining lower MYR concentrations did not alter morphology or the normal cellular functions (Supplementary Figure S1B). Based on the results obtained from the MTT assay, in vitro testing of MYR was limited to 10, 25, 50, 100, 250, and 500 μM respectively.

### 3.3. Transfection & Syncytia Development

**Figure 2:**
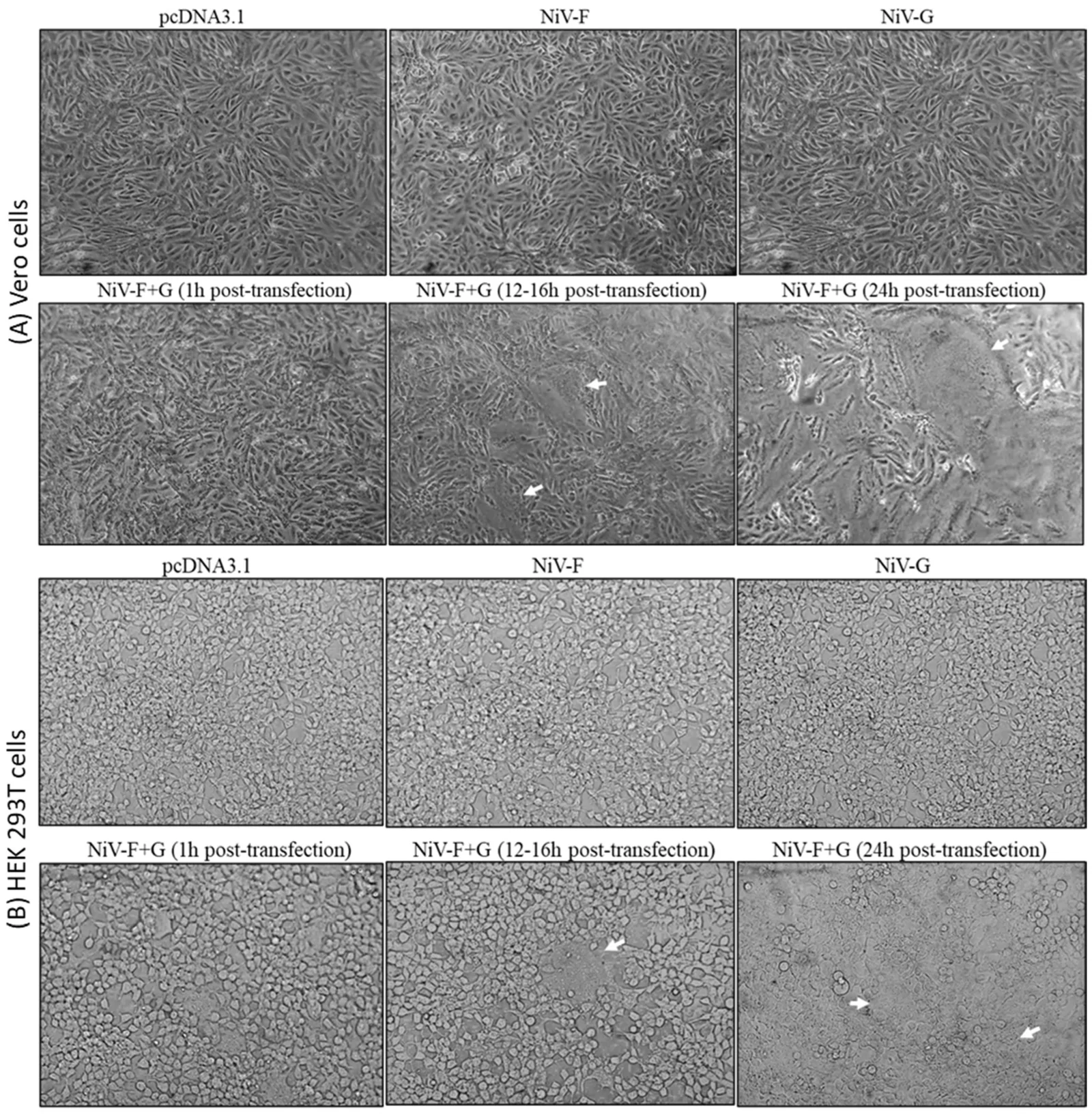
Phase-contrast microscopy images of syncytial development at different time intervals post-transfection with NiV WT F and G plasmids in (**A**) Vero cells (**B**) HEK293T cells. Quantifiable syncytia were observed at 12–16 h post-transfection, while syncytial progression was extensive at 24 h post-transfection. The arrows indicate the presence of syncytia in multi-well cell culture plate.

Quantifiable syncytia were observed in the multi-well plates at 12–18 h post-transfection, whereas 24 h post-transfection, the individual syncytia extensively fused to form giant syncytia. Quantification was carried out 18 h post-transfection. Cells that were co-transfected with NiV F and G developed syncytia and were used as a positive control. Cells transfected with NiV-F alone, G alone, or pcDNA3 did not develop any syncytia and were included as negative controls (Figure 2). No syncytia were observed in non-permissive CHO cells, which therefore served as an additional negative control.

### 3.4. Quantification of Syncytia and Western Blotting

**Figure 3:**
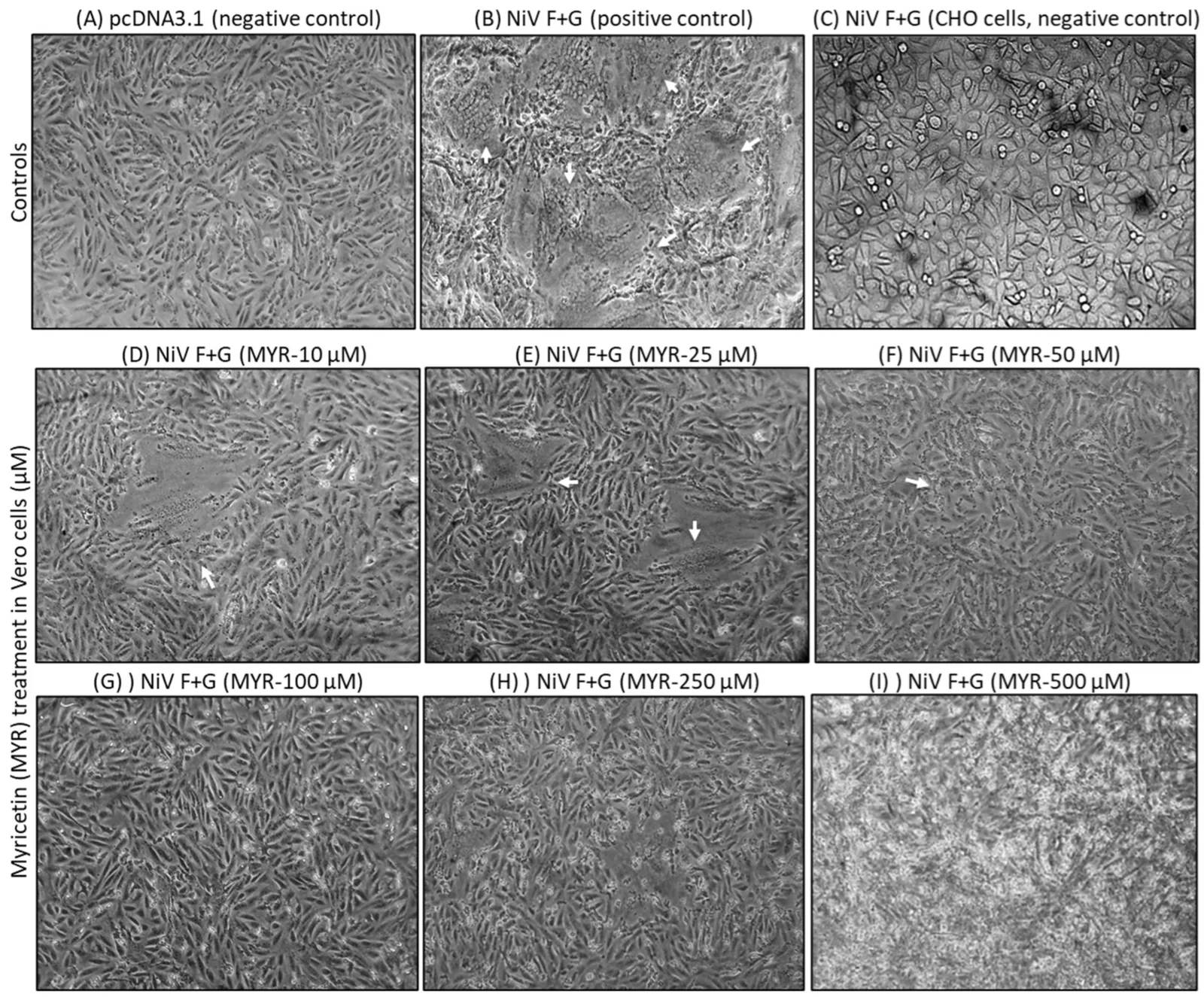
Phase-contrast microscopy images of inhibition of syncytial development (indicated by arrows) in Vero cells by myricetin (MYR) when added at different concentrations 1 h post-transfection with NiV WT F and G plasmids. CHO cells were included as additional negative control. The arrows indicate the presence of syncytia in multi-well cell culture plate.

**Figure 4:**
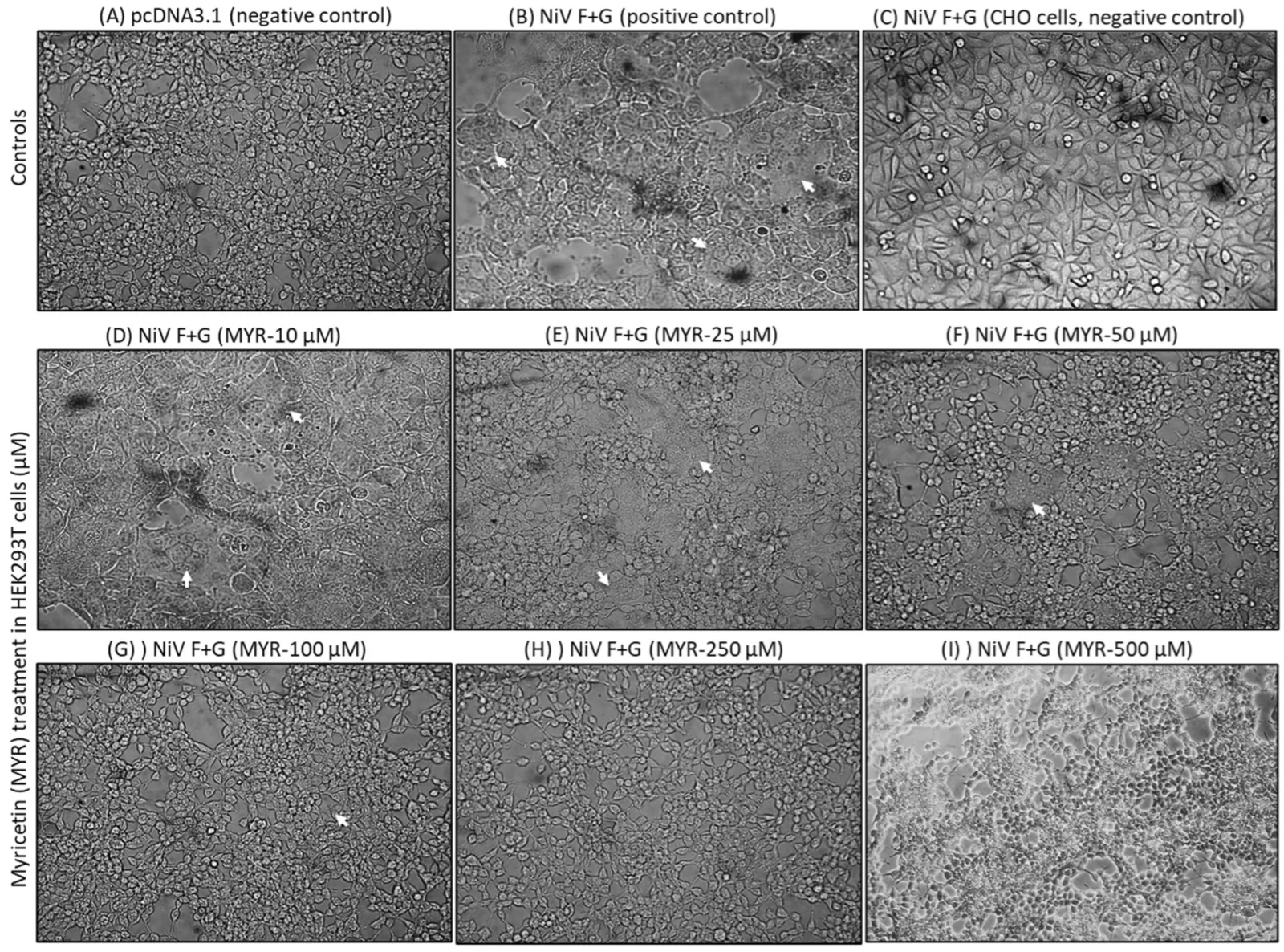
Phase-contrast microscopy images of inhibition of syncytial development (indicated by arrows) in HEK 293T cells by myricetin (MYR) when added at different concentrations 1 h post-transfection with NiV WT F and G plasmids. CHO cells were included as additional negative control. The arrows indicate the presence of syncytia in multi-well cell culture plate.

**Figure 5:**
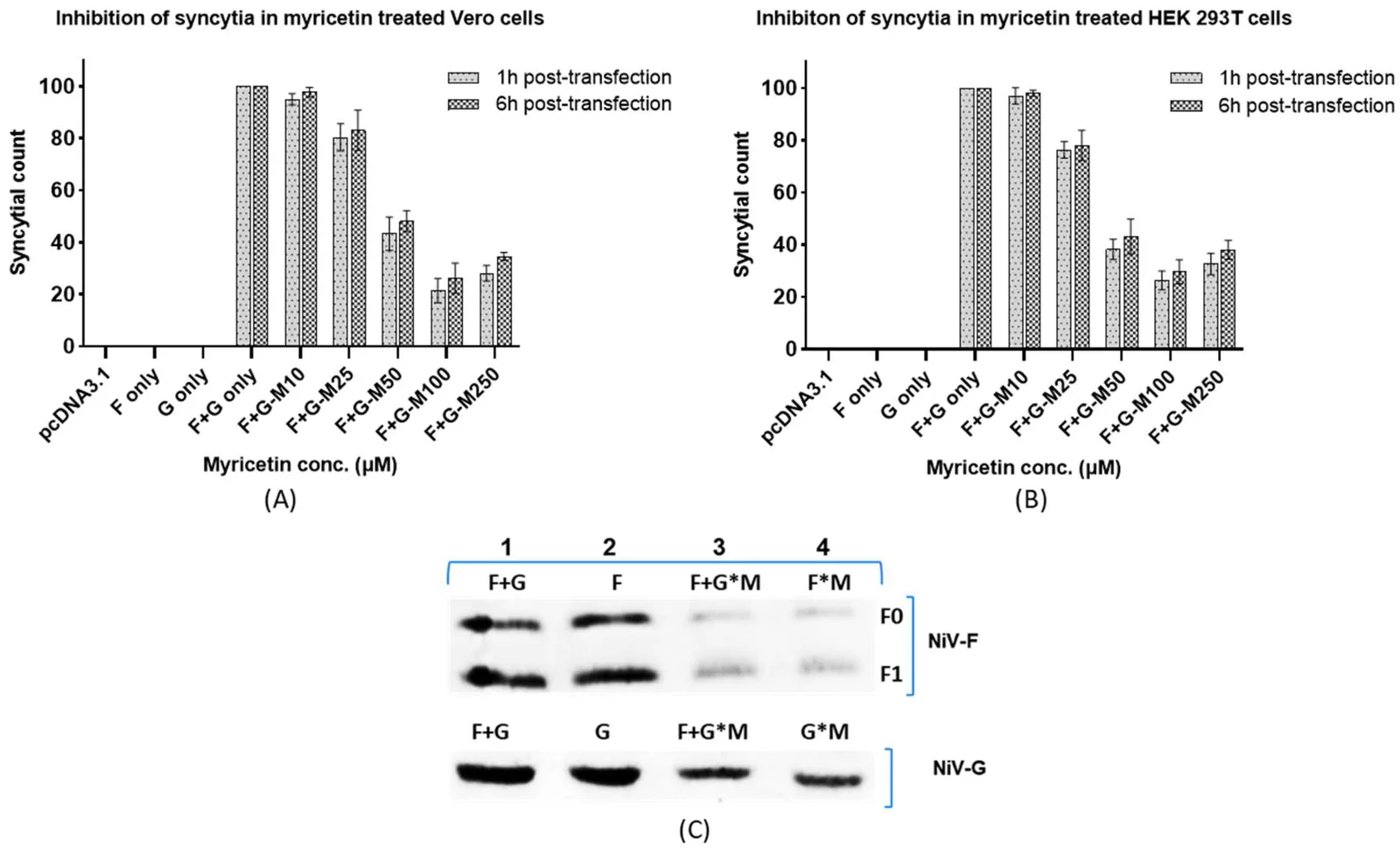
Inhibition of syncytial development by myricetin (MYR) with different concentrations after the addition of MYR post-transfection (**A**) Vero cells (**B**) HEK293T cells. (**C**) Western blot analysis of the cell lysate of HEK 293T cells (transfected with NiV F and G plasmids) with and without MYR (100 μM) addition 1 h post-transfection. (a) Lane-1 (NiV F+G) and Lane-2 (NiV F only) without MYR; Lane-3 (NiV F+G) and Lane-4 (NiV F only) with MYR.

MYR effectively inhibited syncytial development in HEK293T and Vero cells when added 1 h and 6 h following transfection. The percentage of syncytia inhibition was determined by normalizing the syncytial count in positive control wells (F+G without MYR) to 100% and comparing it to the inhibition percentage in MYR-treated HEK and Vero cells. Syncytia were more prominent and measurable in Vero cells compared to HEK293T cells. Though syncytia started to develop 6 h after the transfection, the size and number of syncytia increased during a 12 h period post-transfection. Effective inhibition of syncytia was observed at 50 μM and 100 μM concentration in Vero (Figure 3) and HEK293T (Figure 4). Though the syncytial inhibition was also observed at 250 μM, about 10% of the cells displayed cytotoxicity. The highest percentage of syncytial inhibition (64–80%) was observed in HEK293T and Vero cells when 100 μM MYR was added 1 h following transfection. Syncytial inhibition was prominent when MYR was added 6 h following transfection. However, the overall syncytial count was reduced by 5–10% compared to MYR addition 1 h post-transfection. These results indicated the effectiveness of MYR when added 6 h post-transfection, which allowed sufficient time for the NiV F and G glycoproteins to be expressed in the cells after the transfection (Figure 5A,B). Due to the cytotoxicity of MYR at 500 μM, these results were excluded from the analysis. The protein lysate with and without MYR-treated HEK293T cells were separated by SDS-PAGE gel followed by Western blotting. Compared to the cells without MYR, a marked reduction in the amount of F and G expression was detected in the samples treated with MYR at 100 μM (Figure 5C). These results indicated that MYR possibly interfered with the expression levels of both NiV F and G glycoproteins in vitro.

## 4. Discussion

Studies on the understanding of Nipah virus (NIV) F and G envelope glycoprotein interactions and the syncytial development are of great interest in understanding the pathogenesis of NiV infection towards the development of potential antiviral strategies. In this study, the antiviral potential of MYR was established in two different NiV permissive mammalian cell lines, HEK293T and Vero. The role of MYR in inhibiting the progression of syncytial development was evaluated by different experimental approaches in vitro. These observations are significant given the severity of virus infection in causing severe and acute life-threatening NiV symptoms. Syncytium formation in virus-susceptible cells is the pathological hallmark of NIV infections. NiV infections are known to be characterized by the formation of endothelial syncytia, leading to inflammation and hemorrhage [1,11,34,35,36]. The coordinated action of NiV F and G glycoproteins results in cell-to-cell fusion of adjoining cells, eventually progressing to severe pathological symptoms such as meningitis and even death if untreated. The inhibition of syncytial development demonstrated by MYR in this study showed a dose-dependence (Supplementary Figure S1B). MYR at 500 μM was cytotoxic in all the cell lines tested in this study. At 250 μM, MYR exhibited a ~10% cytotoxicity in HEK293T and Vero cells were also reflected in the quantification of syncytia at higher MYR concentrations. In both the time-of-addition experiments (1 h and 6 h post-transfection), MYR exhibited antiviral potential in inhibiting the syncytial development. A ~5–10% overall reduction in the syncytial count was noticed when MYR was added 6 h post-transfection. These results indicate that MYR was still effective in inhibiting syncytia when added at a later stage after transfecting the cells with NiV F and G. Reduction in the expression levels of NiV F and G glycoproteins in Western blot would likely be attributed to one or several possible roles of MYR: (i) masking key amino acid moieties on the surface of NiV F and G that lead to reduced detection levels; (ii) interfering with the binding affinity of G to ephrinB2/B3 receptors; and (iii) limiting F and G interactions that led to a reduction in syncytial count. In earlier studies, MYR was reported to block Herpes simplex virus (HSV) infection by directly interacting with the viral gD protein. This interaction disrupts HSV gD attachment to host cells and viral fusion with the host cell membrane [27]. MYR played an antiviral role in the inhibition of SARS-CoV-2 via interfering with the virus spike protein from binding to its receptor ACE2 [30]. The antiviral activity of MYR against HIV-1 was determined due to a significant reduction in the binding of HIV-1 virions to SEVI fibrils [37]. Additionally, MYR demonstrated antiviral properties in inhibiting the Infectious bronchitis virus (IBV) replication cycle through the upregulation of the transcription levels in the NF-kB and IRF7 pathways, suggesting a modulating effect of host immune responses inside the cell [17]. Currently, there are no approved therapeutics available for treating NiV infection in humans. A recent study reported that 4′-chloromethyl-2′-deoxy-2′-fluorocytidine (ALS-8112) was found to reduce NiV titer and syncytia formation in vitro [38]. In this study, MYR was effective in inhibiting the syncytial development in HEK293T and Vero cells indicating an antiviral potential against NiV infection in vitro. The findings of this study provide valuable preliminary insights into the antiviral potential of MYR against NiV in vitro. Whether MYR had a specific or stochastic role on the NiV F and G glycoproteins needs further investigation in future studies. The outcomes of this study need to be replicated in other susceptible cell lines and experimental hosts to further the antiviral scope of MYR and derivatives as a preventive line of defense against NiV infections.

## 5. Conclusions

Based on this study, it can be concluded that MYR exhibits effective antiviral potential in inhibiting the syncytial development triggered by NiV F and G glycoproteins in vitro. Further studies are recommended to elucidate the molecular mechanisms underlying the antiviral activity of MYR and its potential applications in in vivo models. 

## Data Availability

The original contributions presented in this study are included in the article/supplementary material. Further inquiries can be directed to the corresponding author.

## Supplementary Materials

The following supporting information can be downloaded at: https://www.mdpi.com/article/10.3390/v17060827/s1, Figure S1: (A) Confirmation of NiV F and G inserts in 1% agarose gel electrophoresis. Lanes 1 and 2: NiV G; lanes 3 and 4: NiV F; M: DNA ladder (B) MTT assay to determine the cell viability of different concentrations of MYR on HEK 293T, Vero and CHO cell lines; Table S1: NiV F and G plasmid DNA combinations tested in HEK 293T, Vero and CHO cells.
