# Supplementary material for: Myricetin Restricts the Syncytial Development Triggered by Nipah Virus Envelope Glycoproteins In Vitro
Source: Viruses. 2025 Jun 7;17(6):827. doi: 10.3390/v17060827 (PMC12197786; doi:10.3390/v17060827)
Supplement: Supplementary file 1 [file viruses-17-00827-s001.zip › viruses-3318740-supplementary.pdf]

viruses

MDPI

Myricetin Restricts the Syncytial Development Triggered by Nipah Virus Envelope Glycoproteins In Vitro

Ananda Murali Rayapati [1], Chanda Chandrasekhar [1], Sudarsana Poojari [2] and Bhadra Murthy Vemulapati [1,2,*]

[1] Koneru Lakshmaiah Education Foundation, Vaddeswaram, Guntur, 522502, India; muralirayapati@gmail.com (A.M.R.); chandrasekharchanda02@gmail.com (C.C.)
[2] Cool Climate Oenology and Viticulture Institute, Brock University, St. Catharines, ON L2S 3A1, Canada; spoojari@brocku.ca (S.P.)
* Correspondence: bvemulapati@brocku.ca (B.M.V.)

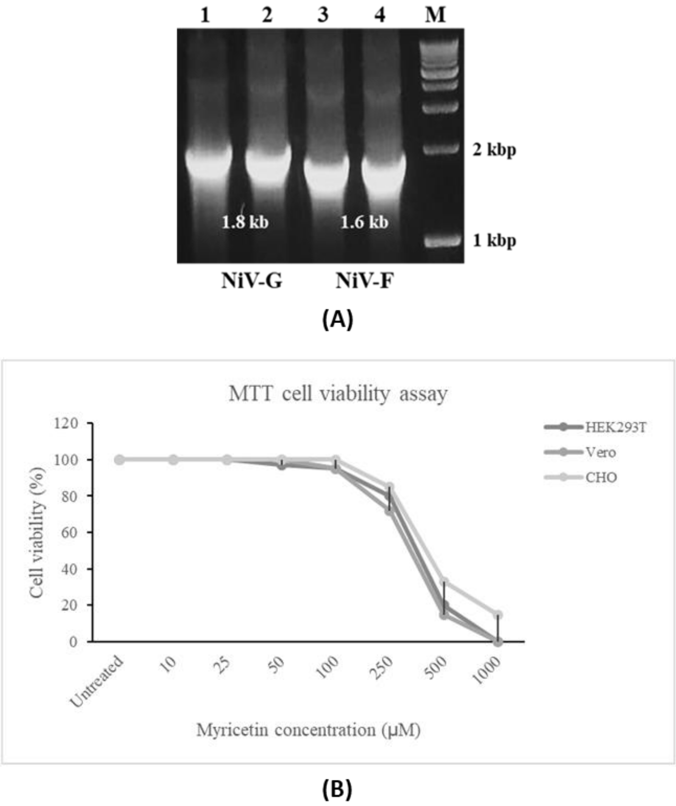

Supplementary Figure S1. (A) Confirmation of NiV F and G inserts in 1% agarose gel electrophoresis. Lanes 1 and 2: NiV G; lanes 3 and 4: NiV F; M: DNA ladder (B) MTT assay to determine the cell viability of different concentrations of MYR on HEK 293T, Vero and CHO cell lines.

Supplementary Table S1. NiV F and G plasmid DNA combinations tested in HEK 293T, Vero and CHO cells.

NiV F and G combinations	***Plasmid DNA ratio***	***Reference in text***
NiV F only	F:pCDNA3.1 (2:1)	Negative control
NiV-G only	G:pCDNA3.1 (1:1)	Negative control
NiV F+G (without myricetin)	F:G (2:1)	Positive control
NiV F+G (with myricetin)	F:G (2:1)	Test samples (quantification of syncyita)
pcDNA3.1 only	pCDNA3.1	Negative control
